# Porcine Epidemic Diarrhea in Europe: In-Detail Analyses of Disease Dynamics and Molecular Epidemiology

**DOI:** 10.3390/v9070177

**Published:** 2017-07-06

**Authors:** Dennis Hanke, Anne Pohlmann, Carola Sauter-Louis, Dirk Höper, Julia Stadler, Mathias Ritzmann, Adi Steinrigl, Bernd-Andreas Schwarz, Valerij Akimkin, Robert Fux, Sandra Blome, Martin Beer

**Affiliations:** 1 Friedrich-Loeffler-Institut, Institute of Diagnostic Virology, D-17493 Greifswald—Insel Riems, Germany; dennis.hanke@fli.de (D.Ha.); anne.pohlmann@fli.de (A.P.); dirk.hoeper@fli.de (D.Hö.); martin.beer@fli.de (M.B.); 2 Friedrich-Loeffler-Institut, Institute of Epidemiology, D-17493 Greifswald—Insel Riems, Germany; carola.sauter-louis@fli.de; 3 Clinic for Swine, Ludwig-Maximilians-University Munich, D-85764 Oberschleissheim, Germany; J.Stadler@med.vetmed.uni-muenchen.de (J.S.); Ritzmann@med.vetmed.uni-muenchen.de (M.R.); 4 Österreichische Agentur für Gesundheit und Ernährungssicherheit GmbH, A-2340 Mödling, Austria; adi.steinrigl@ages.at; 5 Vaxxinova GmbH, Standort Leipzig, D-04103 Leipzig, Germany; bernd-andreas.schwarz@vaxxinova.com; 6 Chemisches und Veterinäruntersuchungsamt Stuttgart, Fellbach, D-70736 Fellbach, Germany; Valerij.Akimkin@cvuas.bwl.de; 7 Institute for Infectious Diseases and Zoonoses, Ludwig-Maximilians-University Munich, D-80539 Munich, Germany; robert.fux@micro.vetmed.uni-muenchen.de

**Keywords:** porcine epidemic diarrhea, porcine epidemic diarrhea virus, phylogenetic analyses, metagenome

## Abstract

Porcine epidemic diarrhea (PED) is an acute and highly contagious enteric disease of swine caused by the eponymous virus (PEDV) which belongs to the genus *Alphacoronavirus* within the *Coronaviridae* virus family. Following the disastrous outbreaks in Asia and the United States, PEDV has been detected also in Europe. In order to better understand the overall situation, the molecular epidemiology, and factors that might influence the most variable disease impact; 40 samples from swine feces were collected from different PED outbreaks in Germany and other European countries and sequenced by shot-gun next-generation sequencing. A total of 38 new PEDV complete coding sequences were generated. When compared on a global scale, all investigated sequences from Central and South-Eastern Europe formed a rather homogeneous PEDV S INDEL cluster, suggesting a recent re-introduction. However, in-detail analyses revealed two new clusters and putative ancestor strains. Based on the available background data, correlations between clusters and location, farm type or clinical presentation could not be established. Additionally, the impact of secondary infections was explored using the metagenomic data sets. While several coinfections were observed, no correlation was found with disease courses. However, in addition to the PEDV genomes, ten complete viral coding sequences from nine different data sets were reconstructed each representing new virus strains. In detail, three pasivirus A strains, two astroviruses, a porcine sapelovirus, a kobuvirus, a porcine torovirus, a posavirus, and an enterobacteria phage were almost fully sequenced.

## 1. Introduction

Porcine epidemic diarrhea (PED) is an acute and highly contagious enteric disease of swine that results in severe enteritis, diarrhea, vomiting, and dehydration. Especially in suckling pigs, mortality can be very high [1]. The causative agent, *Porcine epidemic diarrhea virus* (PEDV), is an enveloped positive single-stranded RNA virus that belongs to genus *Alphacoronavirus* in the family *Coronaviridae* [2].

After its first recognition in the 1970s in Europe [3], the disease caused considerable economic losses, especially in Asia. Since the 1990s, Europe has reported only sporadic cases [4].

In May 2013, a highly virulent PEDV variant emerged in the United States (US), with swine farms experiencing explosive epidemics affecting all age classes of animals, with up to 95% mortality in suckling pigs [5,6]. Apart from these highly virulent strains, apparently less-virulent so-called S INDEL (INDEL standing for insertions and deletions) strains were co-circulating in the US [7,8].

In spring 2014, new PED cases were reported from Germany with different clinical presentations despite a low variability among the detected PEDV strains [9]. It was demonstrated that the causative virus strains were very similar to the S INDEL strains reported in the US. Subsequently, highly similar strains were also found in several European countries, including France [10], Belgium [11], Italy [12], Austria [13], and Spain [14]. At almost the same time, a highly virulent non-INDEL strain was described in Ukraine [15].

Up to now, quite a high number of PED cases have been diagnosed in Germany with a low overall impact on productivity. Varying clinical signs and phenomena such as recurrence of PED in the same herd, or reinfection of pigs that had been affected as piglets, have been observed (field observations reported by veterinary authorities and practitioners).

In an attempt to better understand the overall situation, the molecular epidemiology, and secondary factors with influence on disease severity and dynamics, a detailed study was conducted based on metagenomic datasets obtained from samples of recent PED cases in Germany and Europe. In detail, full-length genomes were generated for phylogenetic analyses, and the role of secondary infections on the observed virulence was explored by investigating the metagenomics datasets. Where possible, epidemiological background information on farm location and type was used to link the data.

## 2. Materials and Methods

### 2.1. Sample Origin

Forty fecal samples (feces or swabs) and intestines were collected from PED cases in Germany, Austria, and Romania between 2014 and 2015 (see Table 1). All samples were derived from routine diagnostic investigations, and were confirmed as PEDV positive by real-time reverse transcription polymerase chain reaction (RT-qPCR) using the assays published by the University of Minnesota Diagnostic Laboratory and Alonso et al. in 2014 [16,17]. The majority of samples (*n* = 35) originated in Germany and were chosen to represent the affected Federal States, different clinical presentations, and pig production sectors (breeding, fattening). In detail, German samples were taken from farms in Baden-Württemberg (*n* = 17), Bavaria (*n* = 4), Lower Saxony (*n* = 5), Schleswig Holstein (*n* = 1), North Rhine-Westphalia (*n* = 4), and Thuringia (*n* = 3). Of these 35 German samples, four had been included in previously reported investigations [9,18], and an additional one had been part of a case report [19]. Four samples were included from Austria [13], and two from Romania. In addition, a historic strain from the virus collection database at the Friedrich-Loeffler-Institute (FLI) was included as reference (strain V215/78, closely related to prototype strain CV777 [20], exact origin unknown).

### 2.2. Sequencing Protocol

#### 2.2.1. Nucleic Acid Extraction

Ribonucleic acids (RNA) were extracted from fecal samples, swabs, or supernatants from homogenized intestines using Trizol Reagent (LifeTechnologies, Darmstadt, Germany) in combination with the RNeasy Mini Kit (Qiagen, Hilden, Germany) and DNase digestion on the spin column. Further concentration and cleaning was done with Agencourt RNAClean XP magnetic beads (Beckman Coulter, Fullerton, CA, USA). The RNA quantity was determined using the Nanodrop ND1000 UV spectrophotometer (Peqlab, Erlangen, Germany).

#### 2.2.2. Synthesis of cDNA, Library Preparation, and Sequencing

The extracted RNA was used as a template for cDNA synthesis with the cDNA Synthesis System (Roche, Mannheim, Germany), and fragmented with a Covaris M220 Focused-ultrasonicator (Covaris, Brighton, UK) applying a target size of 500 bp. The fragmented cDNA was then transformed to barcoded libraries using Illumina compatible adapters (Bioo Scientific Corp., Austin, TX, USA) on a SPRI-TE library system (Beckman Coulter) with SPRIworks Fragment Library Cartridge II (for Roche FLX DNA sequencer; Beckman Coulter) without size selection. Upper and lower size selection was done manually with Agencourt AMPure XP magnetic beads (Beckman Coulter), for a target peak size of 670 bp. The obtained libraries were quality checked with a Bioanalyzer 2100 (Agilent Technologies, Böblingen, Germany) and quantified using the Kapa Library Quantification Kit for Illumina (Kapa Biosystems, Cape Town, South Africa) on a Bio-Rad CFX96 Real-Time System (Bio-Rad Laboratories, Hercules, CA, USA). Sequencing was done with an Illumina MiSeq instrument with MiSeq reagent Kit v3 in 2x300bp PE mode (Illumina, San Diego, CA, USA).

#### 2.2.3. Sequence Assembly and Mapping

Sequence assembly, the subsequent mapping of the raw sequence data, and the analysis of the resulting sequences were done with the Genome Sequencer software suite (v.3.0; Roche) and the Geneious software suite (v.8.1.3; Biomatters Ltd., Auckland, New Zealand). Sequences and their background data were submitted to the European Nucleotide Archive under study accession No. PRJEB19039.

### 2.3. Data Assessment, Generation of Phylogenetic Trees, Metagenomic Analyses

All PEDV sequences generated in this study (see Table S1) and references from the International Nucleotide Sequence Database Collaboration (INSDC) (see Supplementary Table S1) were aligned using ClustalW [21] as implemented in the Geneious software suite. Subsequently, phylogenetic trees were calculated using the maximum likelihood (ML) method as implemented in MEGA (v6.06, [22]) with the best fitting evolutionary model suggested by the program. The General Time Reversible model with Gamma distribution (+G), rate variation (+I) and 1000 bootstrap replicates was applied for the phylogenetic calculations. The resulting trees were visualized in Mega 6.06.

In addition, network analysis was done with European sequences using the sequence of PEDV USA/Indiana12.83/2013 (KJ645635) as outgroup. This strain originated from feeder pigs in Indiana (USA), and was the first S INDEL strain that was detected in 2013 [7]. The resulting network was generated by parsimony split with the software tool SplitsTree (v4.14.2, [23]) by using default settings.

Metagenomic analyses were done with the software RIEMS [24]. The latter allows automated taxonomic classification of reads from metagenomics datasets and helps with the extraction of relevant information from the data pool.

## 3. Results

### 3.1. The New PEDV Strains from Germany, Austria, and Romania Cluster with S INDEL Strains from the USA and Asia

In the context of the present study, 38 new PEDV complete coding sequences were generated and compared with previously published sequences. In detail, 32 complete coding sequences were obtained from German PED cases, four PEDV complete coding sequences were generated from Austrian samples, and two complete coding sequences were assembled analyzing samples from Romanian PED cases. One German sample did not contain sufficiently high numbers of viral sequences to allow generation of a complete coding sequence (L00929 K22/14-01).

**Figure 1:**
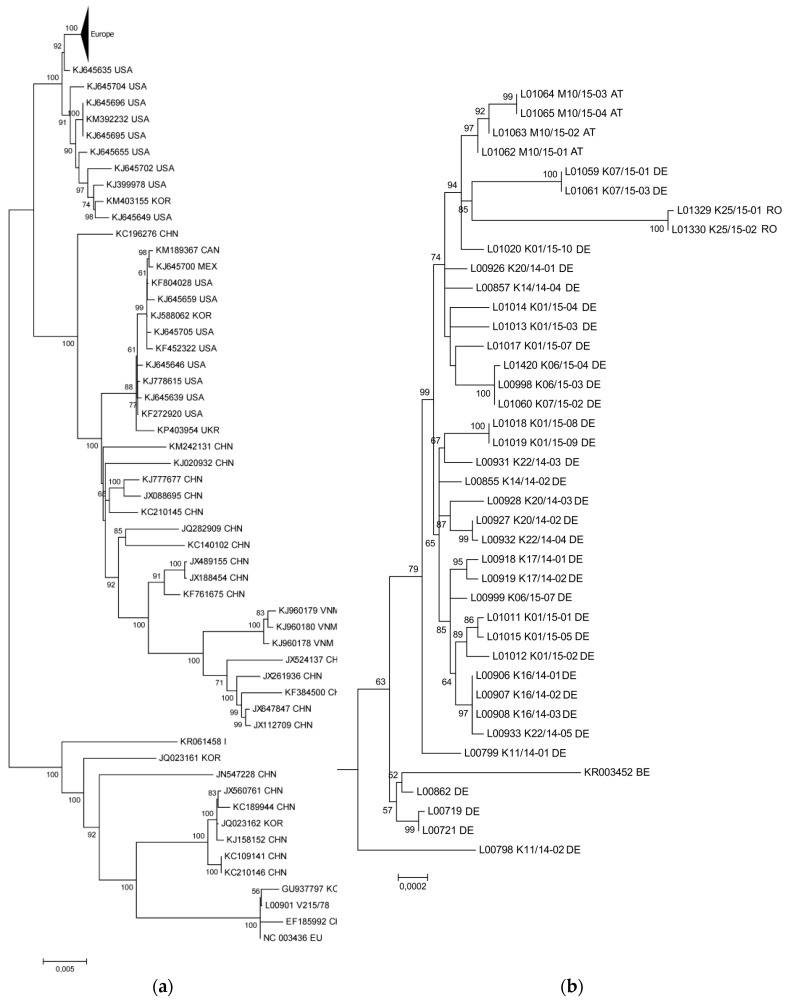
Phylogram generated from 91 PEDV sequences using the Maximum Likelihood algorithm in Mega 6.06 [22]. With General Time Reversible model with Gamma distribution. The phylogram was tested by 1000 bootstrap replicates, branch lengths are measured in the number of substitutions per site (see scale bars). (**a**) European samples in the worldwide context. (**b**) Clustering of European samples. Enlargement of the European cluster (triangle in figure part (**a**)).

In phylogenetic analyses, all sequences from recent PED cases in Germany, Austria, and Romania formed a distinct cluster (see triangle in the upper corner of Figure 1a), which was closely related to S INDEL strains from the USA and Asia (see Figure 1a). Using the described dataset, the prototype S INDEL strain USA/Indiana12.83/2013 (KJ645635) was closest to the European cluster (see Figure 1a). The European strains showed >99.8% overall nucleotide identity with nucleotide substitutions occurring all over the genome, but with a higher density in the S gene. The non-INDEL PEDV strains from the USA and Asia formed their own, more heterogenous cluster (see Figure 1a). The retrospective sample L00901 V215/78 clustered together with CV777 (NC003436) and older Asian strains (SM98, GU937797; LZC, EF185992), and was clearly distinct from all samples representing recent outbreaks.

### 3.2. Phylogenetic Tree and Network Analyses Indicate Segregation into Two Clusters with Intermediate Stages in One Farm

**Figure 2:**
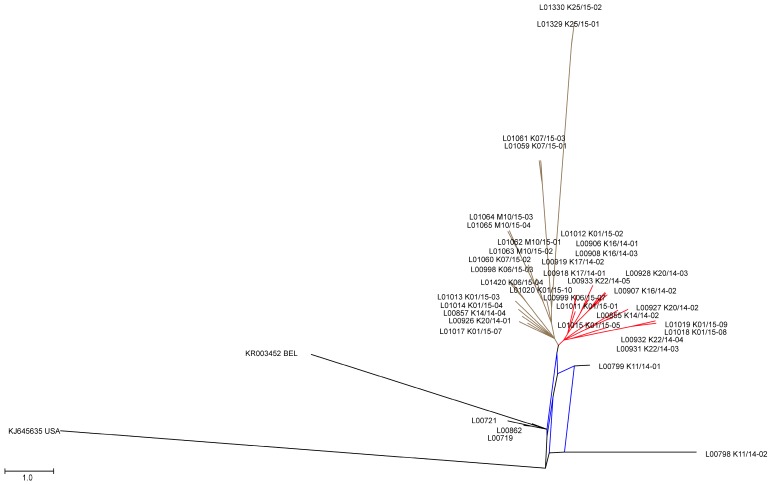
Network Tree generated by parsimony splits with SplitsTree [23] from the European samples of this study using KJ645635 USA as outgroup. The red colored branches show cluster 1, and walnut colored branches cluster 2, respectively. Cluster 3 can be found at the left bottom of the graph (black).

A more detailed phylogenetic analysis of the new European nucleotide sequences by tree (Figure 1b) and network analysis (Figure 2) indicated that the recent European sequences segregated into two major clusters (minor overall variability, see above). Cluster 1 (Figure 2, highlighted in red) consists exclusively of strains from Germany with within cluster sequence identities higher than 99.9%. On the contrary, cluster 2 (Figure 2, walnut) comprised strains from Germany, Austria, and Romania with within cluster sequence identities higher than 99.8%. Five of the sequences under investigation did not fit into one of these clusters. Namely, the viruses from previously published early cases from Germany in 2014 (L00719, L00721; [9,18]) together with the sequence determined for L00862 (corresponding to the outbreaks reported by Henniger and Schwarz [19]) and a Belgian sequence (KR003452) formed a small distinct cluster (cluster 3; see Figure 1b and Figure 2). It is noteworthy that the sequences determined from samples L00798 K11/14-02 and L00799 K11/14-01, which originated from the same farm and were collected at the same date, turned out to differ from one another, and were outliers in the phylogenetic analysis. In the tree generated from the analyzed European sequences, L00798 K11/14-02 took the most remote position, and was closest to the Asian and American strains. The sequence of L00799 K11/14-01, although still an outlier within the European cluster, was closer related to most European strains. From this topology, it appears that L00798 K11/14-02 represents an early introduction but was for some reason not further propagated. On the contrary, L00799 K11/14-01 seems to be progeny of a genotype that was further propagated but itself nevertheless disappeared.

**Figure 3:**
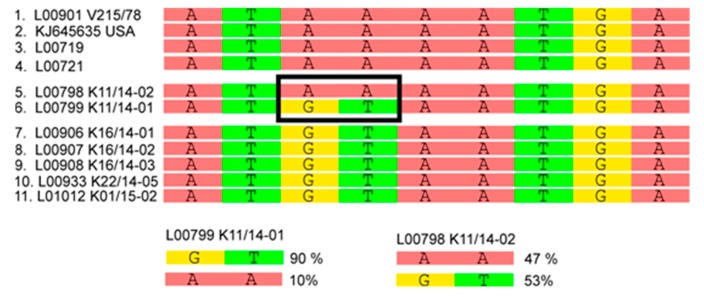
Example of minor variant analysis of two PEDV samples. Boxed nucleotides represent position 27,429-27,430 of strains PEDV/GER/L00798 K11_14-02/2014 and GER/L00799-K11_14-01/2014. These mixtures imply that the two strains represent the transition between different PEDV. More details on the detected variants are given in Supplementary Table S2.

The viruses forming cluster 3 also seem to represent a relatively early genotype but was also not further maintained and hence no additional related sequences were detected. In order to try clarifying the relations, further studies were conducted based on the available next-generation-sequencing data (see Supplementary Table S2). In-depth analyses implied that samples L00798 K11/14-02 and L00799 K11/14-01 are intermediates between the S INDEL strains from the USA (prototype USA/Indiana12.83/2013), the first reported German strains (PEDV/GER/L00719/2014 and PEDV/GER/L00721/2014), and the two new European clusters 1 and 2, respectively. According to this analysis, while L00798 was at the consensus level more closely related to the prototype S INDEL strain, L00799 was closer related to the European strains. Comparison of the single nucleotide frequencies in these two strains showed that both were mixtures of closely related genomes with the corresponding bases still being present as the minority. Variant analyses of these samples revealed differences at 29 nucleotide positions, and moreover, in sample Lib00799 K11/14-02 minor variants at ten different positions (for an example see Figure 3).

### 3.3. Geographic Clustering of PEDV Variants Reveals a Hotspot Region in Baden-Wuerttemberg

**Figure 4:**
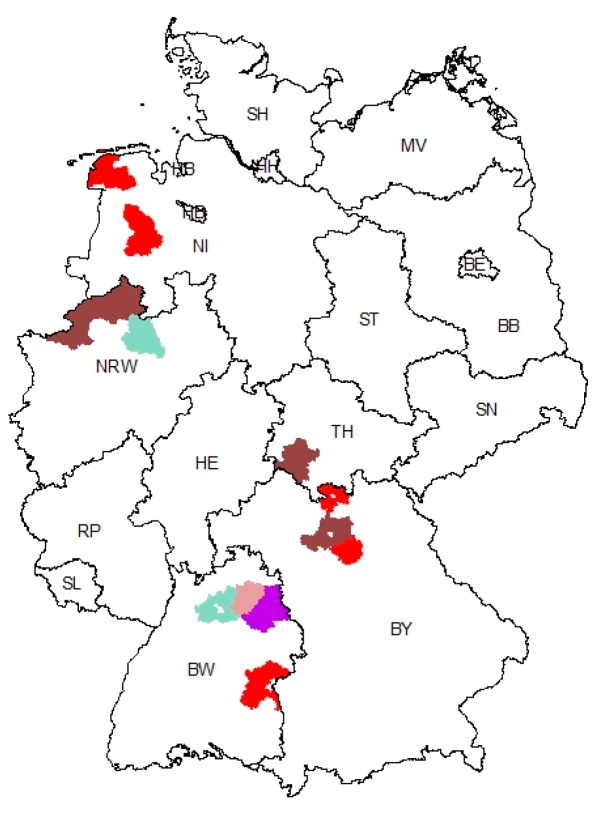
Geographic distribution of PEDV strains in Germany (federal states are presented by the second part of their regional code according to ISO 3166-2). Regions colored red represent the presence of viruses of cluster 1 (parts of Lower Saxony, NI; Bavaria, BY; Baden-Wuerttemberg, BW) and regions colored walnut the presence of viruses of cluster 2 (North Rhine-Westphalia, NRW; Thuringia, TH; Bavaria, BY), respectively. The regions of the first PEDV cases in Europe (Cluster 3) are highlighted light blue/green (border region between NRW and NI, BW). Violet color points to a regional overlap of cluster 1 with ungrouped samples L00798 K11/14-02 and L00799 K11/14-01 (BW).

Phylogeographic investigations did not reveal a correlation between genetic clusters and geographic regions (see Figure 4). Cluster 1 was found in the North-Western part of Germany (Lower Saxony) but also in the Southern Federal States of Bavaria and Baden-Wuerttemberg. The distribution of cluster 2 included the German Federal States of North Rhine-Westphalia, Thuringia, Bavaria, and Baden-Wuerttemberg, as well as Austria and Romania. Cluster 3 was found in the early outbreaks, i.e., at the border of the German Federal States Lower Saxony and North Rhine-Westphalia and in Baden-Wuerttemberg. Apart from the regions with PEDV strains of only one genetic cluster, two regions in the North-Eastern part of Baden-Wuerttemberg showed either clusters 1 and 2 or cluster 1 and the ungrouped sequences (see Figure 4). Close to this region, the first cases were reported, and high outbreak numbers show the high disease impact in this region. Since geographic clustering did not result in a correlation, further available information was taken into account. However, these analyses revealed no correlation between clusters and farm type (production system). Hence, no determinants of the spread of the various genotypes could be elucidated.

### 3.4. Metagenomic Analysis Indicates Several Co-Infections but No Correlation with Disease Severity

In order to explore the impact of possible co-infection patterns on the clinical presentation and outcome of PEDV infections, we analyzed all generated data sets with the metagenomic analysis pipeline RIEMS. Effectively, we found coinfections with bacteria and other viruses in several samples that did not, however, coincide with the different clinical pictures. A unique pattern could not be detected.

For an overview of the bacterial background of selected samples, we compared data sets of duplicates per farm. The most widespread bacteria that could be identified in these samples belonged to *Bacteroidaceae*, *Clostridiaceae*, *Lactobacillaceae*, *Methanobacteriaceae*, *Methanomassiliicoccaceae*, *Porphyromonadaceae* and *Prevotellaceae* (see Supplementary Table S3).

The viruses belonged to the families and orders *Astroviridae*, *Coronaviridae*, *Leviviridae*, *Nidovirales*, *Picornaviridae*, and unclassified *Picornavirales*. Beyond identification of these groups, we successfully reconstructed ten complete viral coding sequences from nine different data sets (see Table 2). In detail, three different Picornaviruses representing new strains of the genus *Pasivirus* were identified, and almost their whole genome information extracted from the datasets. Pasiviruses have already been described as being present in swine [25], but only three full-genome sequences were available in GenBank before now. The new strains are 79.1% to 82.4% identical to the reference sequence Pasivirus A1 (Table 2). Also from the *Picornaviridae* family, a new Porcine sapelovirus (85.4% identical to the reference sequence) and a Posavirus (89.1% identical with the reference in the database) were identified. The genome of a new Porcine torovirus could also be reconstructed as a new member of the Coronavirus family. Porcine toroviruses have been recently identified in PEDV cases from the United States [26] as co-infecting viruses. The complete coding sequence presented here is the third nearly whole genome of this genus. From one of the samples, two other complete coding sequences were derived: a Porcine astrovirus, and a Porcine kobuvirus. In addition, a second Porcine astrovirus genome was completed (complete coding sequence).

## 4. Discussion

Following the disastrous PED outbreaks in the US in 2013, detailed investigations of severe diarrhea cases were performed in several countries including Germany. In spring 2014, this monitoring led to the detection of new PED cases in the North-Western and South-Western parts of Germany [18,19]. The causative virus strains were shown to be closely related and to belong to the so-called S INDEL type of PEDV strains. They were clearly distinct from historic PEDV strains from Europe. In general, these strains seem to be linked with lower overall virulence and lower replication in the host, but these observations need further investigations, especially in direct comparison of S INDEL and non-INDEL strains. In Germany, the clinical picture was highly variable, and did not show any genetic pattern [9]. Following the detection in Germany, similar virus strains were found in several Central European countries, with again little or no impact on productivity. In an attempt to better understand PED disease dynamics and viral evolution in Central Europe, we conducted the presented study based on PEDV complete coding sequences generated from outbreaks between 2014 and 2015. One focus was to study the possible impact of co-infections on disease severity.

We could confirm that the new European PEDVs form a rather homogeneous cluster that is distinct from both the recent highly virulent non-INDEL strains from the US and historic PEDV strains. The fact that the sequence identities within the cluster are still high, and that those strains were not observed in Europe until 2014 [12], strengthens the idea of a single or at least simultaneous introduction into Central Europe. Our phylogenetic network analyses revealed two new clusters which are different from the first European cases. So far however, no correlation between genetic clusters and location, disease severity, or farm type could be established. Interestingly, we also identified two samples, L00799 K11/14-01 and L00798 K11/14-02, respectively, that are branches separated from previous PEDV strains. It is tempting to speculate that these samples are directly related to one of the first new European PEDV-strains (GER/L00719/2014, GER/L00721/2014). Samples L00799 K11/14-01 and L00798 K11/14-02 were collected from the same farm in the North-Eastern part of the German Federal State of Baden-Wuerttemberg at the same time. In this region, samples from all PEDV clusters were detected. By analyzing the variants that were found in both samples in detail, we identified an identical consensus sequence with a broad minor variant virus population. Some of these variants can also be found in older PEDV strains, such as CV777, the US S INDEL viruses (e.g., KJ645,635), and the first described European PEDV (L00719, L00721). Other variants were detected in the new PEDV clusters. It is therefore not unlikely that the “hotspot” region in Baden-Wuerttemberg, where all PEDV clusters were detected, is the place of the disease’s introduction into Germany. At least, the possible connection of these strains with the origin of the novel PEDV strains is highlighted.

In order to investigate the impact of coinfections on clinical outcome and mortality rates, we performed metagenomic analyses from the sequenced fecal samples. We identified coinfections with several other viruses, and subsequently extracted complete coding sequences from the data sets. All these viruses were also found in previous studies of swine feces [27], but could not be identified as a cofactor of the high mortality. Moreover, the analysis of the bacterial community revealed specific patterns for individual holdings, but also provided no clear evidence if a special pattern of bacterial coinfection could cause the high mortality in those animals. Recently, Koh, et al. [28] identified changes of the swine gut microbiota in an experimental trial in response to PEDV infection. Nevertheless, in our study we could not verify these findings in samples from the field, as we identified clearly different patterns of organisms in the microbial communities. Furthermore, interesting viral sequences were detected, but also here no characteristic pattern of viral coinfections could be identified.

The influence of coinfections on the clinical outcome of PEDV infections is therefore highly complex and still not understood.

## 5. Conclusions

In summary, our data strengthen the hypothesis of a single or simultaneous introduction of PEDV into Germany and Central Europe in 2014. Despite the fact that the virus strains are still closely related, two major clusters were now identified using phylogenetic network analyses. Our finding of a sample set at an intermediate stage allowed a first insight into virus evolution, and it is tempting to speculate that the respective region in Baden-Wuerttemberg is a hotspot for PEDV introduction and evolution. Further epidemiological investigations into field epidemiology are needed to better understand trade or product related patterns that could help to understand both dynamics and possible routes of introduction.

Furthermore, in order to uncover putative co-infections that could explain the different clinical outcome of infections with very similar PEDV strains, we performed comprehensive metagenomic analyses. Albeit that numerous coinfections are present in the analyzed samples, none of these could be identified as a major factor for the clinical outcome. Additional studies will be needed to find a context and link between low and high mortality caused by the same virus.

## Figures and Tables

**Table 1:** Details and metadata of samples investigated in this study.

Sample ID	Country	Federal State	Comments
L00719 BH76/14_1	Germany	Baden-Wuerttemberg	Fecal material from fattening pigs, sample referred to as farm 4 in metagenomic analyses
L00721 BH76/14_2	Germany	Baden-Wuerttemberg	Fecal material from fattening pigs, referred to as farm 4 in metagenomic analyses
L00798 K11/14-02	Germany	Baden-Wuerttemberg	Fecal material, sample referred to as farm 5 in metagenomic analyses
L00799 K11/14-01	Germany	Baden-Wuerttemberg	Fecal material, sample referred to as farm 5 in metagenomic analyses
L00855 K14/14-02	Germany	Baden-Wuerttemberg	Fecal material from piglets and weaners, referred to as farm 2 in metagenomic analyses
L00857 K14/14-04	Germany	Baden-Wuerttemberg	Fecal material from fattening pigs
L00862	Germany	Border Lower-Saxony North Rhine-Westphalia	Nucleic acids derived from fecal material of fattening pigs
L00901 V215/78	Germany	not provided	Cell culture virus, closely related to CV777 [20] from 1978
L00906 K16/14-01	Germany	Baden-Wuerttemberg	Fecal material from a sow, high mortality in piglets, referred to as farm 6
L00907 K16/14-02	Germany	Baden-Wuerttemberg	Fecal material from a sow and their suckling piglets, high mortality in piglets, referred to as farm 6
L00908 K16/14-03	Germany	Baden-Wuerttemberg	Fecal material from a sow and their suckling piglets, high mortality in piglets, referred to as farm 6
L00918 K17/14-01	Germany	Baden-Wuerttemberg	Fecal material from a sow, referred to as farm 3
L00,919 K17/14-02	Germany	Baden-Wuerttemberg	Fecal material from suckling pigs, referred to as farm 3
L00926 K20/14-01	Germany	Bavaria	No details provided
L00927 K20/14-02	Germany	Baden-Wurttemberg	No details provided
L00928 K20/14-03	Germany	Baden-Wuerttemberg	No details provided
L00929 K22/14-01	Germany	Baden-Wuerttemberg	Re-introduction into farm 2, no complete PEDV genome
L00931 K22/14-03	Germany	Baden-Wuerttemberg	No details provided
L00932 K22/14-04	Germany	Baden-Wuerttemberg	No details provided
L00933 K22/14-05	Germany	Baden-Wuerttemberg	No details provided
L00998 K06/15-03	Germany	North Rhine-Westphalia	Fecal material from sows, high mortality in suckling pigs, referred to as farm 1
L00999 K06/15-07	Germany	Schleswig-Holstein	Fecal material from fattening pigs
L01011 K01/15-01	Germany	Lower Saxony	Fecal material from a farm with affected fattening and breeding animals
L01012 K01/15-02	Germany	Lower Saxony	Fecal material from fattening pigs
L01013 K01/15-03	Germany	North Rhine-Westphalia	Fecal material from fattening pigs
L01014 K01/15-04	Germany	North Rhine-Westphalia	Fecal material from fattening pigs
L01015 K01/15-05	Germany	Lower Saxony	Fecal material from a farm with affected fattening and breeding animals
L01017 K01/15-07	Germany	Bavaria	Fecal material from gilts
L01018 K01/15-08	Germany	Bavaria	Fecal material from fattening pigs
L01019 K01/15-09	Germany	Bavaria	Fecal material from fattening pigs
L01020 K01/15-10	Germany	Thuringia	Fecal material from a farm with affected fattening and breeding animals
L01059 K07/15-01	Germany	Thuringia	Fecal material from young fattening pigs that had been affected by PED as suckling pigs
L01060 K07/15-02	Germany	North Rhine-Westphalia	Fecal material from a sow and their piglets, high mortality in suckling pigs, corresponds to farm 1
L01061 K07/15-03	Germany	Thuringia	Fecal material from fattening pigs, possible re-infection
L01062 M10/15-01	Austria	Upper Austria	Fecal material from fattening pigs
L01063 M10/15-02	Austria	Upper Austria	Fecal material from fattening pigs
L01064 M10/15-03	Austria	Upper Austria	Fecal material from fattening pigs
L01065 M10/15-04	Austria	Upper Austria	Fecal material from fattening pigs
L01329 K25/15-01	Romania	not applicable	No details provided
L01330 K25/15-02	Romania	not applicable	No details provided
L01420 K06/15-04	Germany	North Rhine-Westphalia	Fecal material from fattening pigs, corresponds to farm 1 (high piglet mortality)

PED = porcine epidemic diarrhea; PEDV = PED virus.

**Table 2:** Complete coding sequences of viruses discovered as coinfection in feces from PEDV infected swines.

Sample ID	Virus
L00721 Pa	Pasivirus A
L00798 K11/14-02 S	Porcine sapelovirus A
L00799 K11/14-01 Pa	Pasivirus A
L00855 K14/14-02 A	Porcine astrovirus
L00919 K17/14-02 A	Porcine astrovirus
L00919 K17/14-02 K	Porcine kobuvirus
L00926 K20/14-01 T	Porcine torovirus
L00928 K20/14-03 E	Enterobacteria Phage MS2
L01017 K01/15-07 Po	Posavirus 1
L01061 K07/15-03 Pa	Pasivirus A

## Supplementary Materials

The following are available online at www.mdpi.com/1999-4915/9/7/177/s1, Table S1: Overview of the sequences used as references for phylogenetic analyses, Table S2: Nucleotides minor variants of sequenced PEDV samples, Table S3: Overview of metagenome analysis of selected PEDV samples.
