# Supplementary material for: Porcine Epidemic Diarrhea in Europe: In-Detail Analyses of Disease Dynamics and Molecular Epidemiology
Source: Viruses. 2017 Jul 6;9(7):177. doi: 10.3390/v9070177 (PMC5537669; doi:10.3390/v9070177)
Supplement: Supplementary file 1 [file viruses-09-00177-s001.pdf]

Supplementary Table S1: Overview of the sequences used as references for phylogenetic analyses.

Sample ID	Location	GenBank accession No.	Reference
EF185992 CHN	China	EF185992	Cheng et al. (2006) (unpublished)
GU937797 KOR	South Korea	GU937797	Cha et a. (2010) (unpublished)
JN547228 CHN	China	JN547228	J. Chen et al. (2011)
JQ023161 KOR	South Korea	JQ023161	Park et al. (2012)
JQ023162 KOR	South Korea	JQ023162	Park et al. (2012)
JQ282909 CHN	China	JQ282909	J. Chen et al. (2012)
JX088695 CHN	China	JX088695	Luo et al. (2012)
JX112709 CHN	China	JX112709	Fan et al. (2012)
JX188454 CHN	China	JX188454	Bi et al. (2012)
JX261936 CHN	China	JX261936	X. Tian et al. (2012) (direct submission)
JX489155 CHN	China	JX489155	F. Chen et al. (2012)
JX524137 CHN	China	JX524137	Zhou et al. (2012)
JX560761 CHN	China	JX560761	Zhao et al. (2012)
JX647847 CHN	China	JX647847	Wei et al. (2012)
KC109141 CHN	China	KC109141	Li et al. (2013)
KC140102 CHN	China	KC140102	F. Chen et al. (2012 (unpublished)
KC189944 CHN	China	KC189944	X. Wang et al. (2012) (unpublished)
KC196276 CHN	China	KC196276	X. M. Wang (2013)
KC210145 CHN	China	KC210145	Liu et al. (2012) (direct submission)
KC210146 CHN	China	KC210146	Liu et al. (2012) (direct submission)
KF272920 USA	USA	KF272920	Marthaler et al. (2013)
KF384500 CHN	China	KF384500	Y. Tian et al. (2013)
KF452322 USA	USA	KF452322	Stevenson et al. (2013)
KF761675 CHN	China	KF761675	Wan et al. (2013) (unpublished)
KF804028 USA	USA	KF804028	H. Huang et al. (2013)
KJ020932 CHN	China	KJ020932	Jia et al. (2014)
KJ158152 CHN	China	KJ158152	Ku et al. (2014) (unpublished)
KJ399978 USA	USA	KJ399978	L. Wang et al. (2014)
KJ588062 KOR	South Korea	KJ588062	Choi et al. (2014)
KJ645635 USA	USA	KJ645635	Vlasova et al. (2014)
KJ645639 USA	USA	KJ645639	Vlasova et al. (2014)
KJ645646 USA	USA	KJ645646	Vlasova et al. (2014)
KJ645649 USA	USA	KJ645649	Vlasova et al. (2014)
KJ645655 USA	USA	KJ645655	Vlasova et al. (2014)
KJ645659 USA	USA	KJ645659	Vlasova et al. (2014)
KJ645695 USA	USA	KJ645695	Vlasova et al. (2014)
KJ645696 USA	USA	KJ645696	Vlasova et al. (2014)
KJ645700 MEX	MEX	KJ645700	Vlasova et al. (2014)
KJ645702 USA	USA	KJ645702	Vlasova et al. (2014)
KJ645704 USA	USA	KJ645704	Vlasova et al. (2014)
KJ645705 USA	USA	KJ645705	Vlasova et al. (2014)
KJ777677 CHN	China	KJ777677	H. Zhou et al. (2014) (unpublished)
KJ778615 USA	USA	KJ778615	Lawrence et al. (2014)

KJ960178 VNM	Vietnam	KJ960178	Vui et al. (2014)
KJ960179 VNM	Vietnam	KJ960179	Vui et al. (2014)
KJ960180 VNM	Vietnam	KJ960180	Vui et al. (2014)
KM189367 CAN	Canada	KM189367	Pasick et al. (2014)
KM242131 CHN	China	KM242131	Song et al. (2014)
KM392232 USA	USA	KM392232	Oka et al. (2014)
KM403155 KOR	South Korea	KM403155	Lee et al. (2014)
KP403954 UKR	Ukraine	KP403954	Dastjerdi et al. (2015) (unpublished)
KR003452 BEL	Belgium	KR003452	Theuns et al. (2015)
KR061458 I	Italy	KR061458	Boniotti et al. (2016)
L00719	Europe	LM645058	Hanke et al. (2015)
L00721	Europe	LM645057	Hanke et al. (2015)
NC003436 EU	Europe	NC003436	Kocherhans et al. (2001)

Supplementary Table S2: Nucleotide minor variants of sequenced PEDV samples. First and last base positions refer to the complete coding sequence of the respective strain.

Sequence Name	Nucleotide (original)	Nucleotide (variant)	Frequency (%)	First base position	Last base position	First base position (with gaps)	Last base position (with gaps)
L00798 K11/14-02	T	G	21	19.345	19.345	19.345	19.345
L00798 K11/14-02	T	G	26	19.461	19.461	19.461	19.461
L00798 K11/14-02	G	A	19	19.998	19.998	19.998	19.998
L00798 K11/14-02	G	A	21	21.009	21.009	21.021	21.021
L00798 K11/14-02	C	T	19	21.149	21.149	21.164	21.164
L00798 K11/14-02	A	T	14	21.220	21.220	21.235	21.235
L00798 K11/14-02	C	T	14	21.340	21.340	21.355	21.355
L00798 K11/14-02	C	A	23	21.535	21.535	21.550	21.550
L00798 K11/14-02	C	T	26	21.686	21.686	21.710	21.710
L00798 K11/14-02	G	A	27	21.705	21.705	21.729	21.729
L00798 K11/14-02	T	C	19	21.994	21.994	22.018	22.018
L00798 K11/14-02	C	T	28	22.214	22.214	22.238	22.238
L00798 K11/14-02	T	C	23	22.349	22.349	22.373	22.373
L00798 K11/14-02	C	T	17	23.063	23.063	23.087	23.087
L00798 K11/14-02	T	C	11	23.267	23.267	23.291	23.291
L00798 K11/14-02	T	C	15	23.570	23.570	23.594	23.594
L00798 K11/14-02	T	C	13	23.720	23.720	23.744	23.744
L00798 K11/14-02	T	G	21	23.919	23.919	23.943	23.943
L00798 K11/14-02	C	T	18	24.050	24.050	24.074	24.074
L00798 K11/14-02	G	A	14	24.114	24.114	24.138	24.138
L00798 K11/14-02	C	T	19	24.719	24.719	24.743	24.743
L00798 K11/14-02	C	A	20	25.037	25.037	25.061	25.061
L00798 K11/14-02	C	T	20	27.193	27.193	27.217	27.217
L00798 K11/14-02	AA	GT	10	27.429	27.430	27.453	27.454

L00798 K11/14-02	A	T	25	27.704	27.704	27.728	27.728
L00798 K11/14-02	A	C	25	27.728	27.728	27.752	27.752
L00798 K11/14-02	T	C	25	27.772	27.772	27.796	27.796
L00799 K11/14-01	A	G	13	3.302	3.302	3.302	3.302
L00799 K11/14-01	G	A	13	3.435	3.435	3.435	3.435
L00799 K11/14-01	T	C	10	3.619	3.619	3.619	3.619
L00799 K11/14-01	T	C	10	3.971	3.971	3.971	3.971
L00799 K11/14-01	G	T	11	4.241	4.241	4.241	4.241
L00799 K11/14-01	G	T	15	4.423	4.423	4.423	4.423
L00799 K11/14-01	C	T	10	5.203	5.203	5.203	5.203
L00799 K11/14-01	C	T	12	5.293	5.293	5.293	5.293
L00799 K11/14-01	T	C	12	5.875	5.875	5.875	5.875
L00799 K11/14-01	T	C	34	6.405	6.405	6.405	6.405
L00799 K11/14-01	G	T	28	19.345	19.345	19.345	19.345
L00799 K11/14-01	G	T	25	19.461	19.461	19.461	19.461
L00799 K11/14-01	A	G	26	19.998	19.998	19.998	19.998
L00799 K11/14-01	A	G	32	21.010	21.010	21.022	21.022
L00799 K11/14-01	T	C	28	21.149	21.149	21.164	21.164
L00799 K11/14-01	C	T	31	21.179	21.179	21.194	21.194
L00799 K11/14-01	T	A	37	21.220	21.220	21.235	21.235
L00799 K11/14-01	T	C	40	21.340	21.340	21.355	21.355
L00799 K11/14-01	A	C	26	21.535	21.535	21.550	21.550
L00799 K11/14-01	T	C	21	21.686	21.686	21.710	21.710
L00799 K11/14-01	A	G	22	21.705	21.705	21.729	21.729
L00799 K11/14-01	C	T	30	21.994	21.994	22.018	22.018
L00799 K11/14-01	T	T	25	22.214	22.214	22.238	22.238
L00799 K11/14-01	C	T	26	22.349	22.349	22.373	22.373
L00799 K11/14-01	T	C	28	23.063	23.063	23.087	23.087
L00799 K11/14-01	C	T	43	23.267	23.267	23.291	23.291

L00799 K11/14-01	C	T	35	23.570	23.570	23.594	23.594
L00799 K11/14-01	C	T	45	23.720	23.720	23.744	23.744
L00799 K11/14-01	G	T	31	23.919	23.919	23.943	23.943
L00799 K11/14-01	T	C	32	24.050	24.050	24.074	24.074
L00799 K11/14-01	A	G	40	24.114	24.114	24.138	24.138
L00799 K11/14-01	T	C	31	24.719	24.719	24.743	24.743
L00799 K11/14-01	A	C	32	25.037	25.037	25.061	25.061
L00799 K11/14-01	T	C	37	27.193	27.193	27.217	27.217
L00799 K11/14-01	GT	AA	47	27.429	27.430	27.453	27.454
L00799 K11/14-01	T	A	24	27.704	27.704	27.728	27.728
L00799 K11/14-01	C	A	24	27.728	27.728	27.752	27.752
L00799 K11/14-01	C	T	23	27.772	27.772	27.796	27.796
L00857 K14/14-04	T	C	11	2.589	2.589	2.608	2.608
L00857 K14/14-04	T	G	10	3.304	3.304	3.323	3.323
L00857 K14/14-04	T	C	11	16.096	16.096	16.115	16.115
L00857 K14/14-04	C	T	11	22.126	22.126	22.169	22.169
L00857 K14/14-04	T	C	14	22.533	22.533	22.576	22.576
L00901 V215/78	-	TTTTGAAAAGGTCCACGTGCAGTGATGTTTCTT GGACTTTTTCAATACACGA	5-10	24.754	24.753	24.785	24.836
L00918 K17/14-01	G	A	41	18.585	18.585	18.608	18.608
L00918 K17/14-01	A	G	45	21.076	21.076	21.114	21.114
L00928 K20/14-03	T	C	21	6.835	6.835	6.835	6.835
L00928 K20/14-03	ATGCTA	-	10	20.974	20.979	20.986	20.991
L00928 K20/14-03	C	T	33	22.135	22.135	22.159	22.159
L00928 K20/14-03	C	T	11	22.582	22.582	22.606	22.606
L00932 K22/14-04	T	C	24	10.510	10.510	10.510	10.510
L00932 K22/14-04	A	G	20	23.972	23.972	23.996	23.996
L00932 K22/14-04	T	C	17	25.077	25.077	25.101	25.101
L00933 K22/14-05	A	G	11	4.520	4.520	4.520	4.520

L00933 K22/14-05	C	T	10	8.065	8.065	8.065	8.065
L00933 K22/14-05	G	A	30	21.099	21.099	21.114	21.114
L00933 K22/14-05	A	G	19	21.100	21.100	21.115	21.115
L01015 K01/15-05	T	C	42	424	424	424	424
L01015 K01/15-05	G	A	35	1.737	1.737	1.737	1.737
L01015 K01/15-05	C	A	37	1.759	1.759	1.759	1.759
L01015 K01/15-05	T	C	36	2.334	2.334	2.334	2.334
L01015 K01/15-05	T	C	40	2.821	2.821	2.821	2.821
L01015 K01/15-05	G	T	50	5.530	5.530	5.530	5.530
L01015 K01/15-05	G	A	48	6.814	6.814	6.814	6.814
L01015 K01/15-05	T	C	34	22.790	22.790	22.814	22.814
L01017 K01/15-07	T	C	24	7.322	7.322	7.339	7.339
L01017 K01/15-07	G	A	12	21.082	21.082	21.114	21.114
L01017 K01/15-07	C	A	23	22.354	22.354	22.395	22.395
L01017 K01/15-07	C	T	12	22.510	22.510	22.551	22.551
L01017 K01/15-07	G	T	15	24.279	24.279	24.320	24.320
L01019 K01/15-09	C	T	17	5.548	5.548	5.548	5.548
L01019 K01/15-09	T	C	15	19.953	19.953	19.953	19.953
L01019 K01/15-09	C	A	25	22.371	22.371	22.395	22.395
L01020 K01/15-10	T	C	26	21.179	21.179	21.194	21.194
L01059 K07/15-01	G	T	21	25.233	25.233	25.257	25.257
L01061 K07/15-03	C	T	11	20.864	20.864	20.876	20.876
L01061 K07/15-03	G	T	23	25.233	25.233	25.257	25.257
L01063 M10/15-02	G	T	47	21.878	21.878	21.902	21.902
L01063 M10/15-02	C	A	22	24.744	24.744	24.768	24.768
L01064 M10/15-03	T	C	12	5.965	5.965	5.965	5.965
L01065 M10/15-04	G	T	39	1.709	1.709	1.709	1.709
L01065 M10/15-04	T	C	32	2.723	2.723	2.723	2.723
L01065 M10/15-04	T	G	30	4.672	4.672	4.672	4.672

L01065 M10/15-04	T	C	14	24.074	24.074	24.098	24.098
L01329 K25/15-01	T	C	44	532	532	544	544
L01329 K25/15-01	C	T	50	1.408	1.408	1.420	1.420
L01329 K25/15-01	C	T	48	2.491	2.491	2.503	2.503
L01329 K25/15-01	T	C	45	22.055	22.055	22.091	22.091
L01329 K25/15-01	G	T	12	23.087	23.087	23.123	23.123
L01420 K06/15-04	T	C	12	7.145	7.145	7.145	7.145
L01420 K06/15-04	C	T	12	15.430	15.430	15.430	15.430
L01420 K06/15-04	C	T	11	20.592	20.592	20.592	20.592
L01420 K06/15-04	T	C	32	27.716	27.716	27.740	27.740

Supplementary Table S3: Overview of metagenome analysis of selected PEDV samples.

Organism name (family)	Farm 1			Farm 2		Farm 3		Farm 4		Farm 5		Farm 6		
	L00998 K06/15-03	L01060 K07/15-02	L01420 K06/15-04	L00855 K14/14-02	L00929 K22/14-01	L00918 K17/14-01	L00919 K17/14-02	L00719	L00721	L00798 K11/14-02	L00799 K11/14-01	L00906 K16/14-01	L00907 K16/14-02	L00908 K16/14-03
Acidaminococcaceae	-	+	+	-	-	-	-	+	-	-	+	-	-	-
Alicyclobacillaceae	-	-	-	-	-	-	-	-	-	-	-	-	+	-
Amphileptidae	-	-	-	-	-	-	-	-	+	-	-	-	-	-
Astroviridae	-	-	-	+	-	-	+	-	-	-	-	-	-	-
Bacillaceae	-	-	+	-	-	+	-	+	-	+	+	+	+	-
Bacteroidaceae	+	+	+	+	+	+	+	+	+	+	+	-	+	+
Brachyspiraceae	-	-	-	+	-	-	-	-	-	+	-	-	-	-
Campylobacteraceae	+	+	+	+	+	-	-	-	+	+	+	-	+	+
Carnobacteriaceae	-	-	+	-	-	+	-	-	-	+	-	+	+	-
Chlamydiaceae	-	-	-	-	-	-	-	-	-	-	+	-	-	-
Clostridiaceae	+	+	+	+	+	+	-	+	+	+	+	+	+	+
Comamonadaceae	-	-	-	-	-	-	+	-	-	-	-	-	-	-
Cordycipitaceae	-	-	-	-	-	-	+	-	-	-	-	-	+	-
Coriobacteriaceae	-	-	-	-	-	-	-	+	-	-	-	-	-	-
Coronaviridae	+	-	+	+	-	+	+	+	+	+	+	+	+	+
Corynebacteriaceae	-	-	-	-	-	-	+	-	-	-	-	-	-	-
Cryptosporidiidae	-	-	-	-	-	-	-	+	-	-	-	-	-	-
Cyclobacteriaceae	-	+	-	-	-	-	-	-	-	-	-	-	-	-
Dipodascaceae	-	-	-	-	-	-	-	-	-	-	-	+	-	-
Enterobacteriaceae	-	+	-	+	+	+	+	-	-	+	+	+	+	+
Enterococcaceae	-	-	+	-	-	-	-	+	-	+	-	+	-	-
Erysipelotrichaceae	-	-	-	+	+	-	-	+	-	+	+	-	+	-
Eubacteriaceae	-	-	-	+	-	-	-	+	+	+	+	-	-	-
Flavobacteriaceae	-	+	+	-	+	-	-	+	+	+	+	-	+	-
Fusobacteriaceae	-	-	-	+	-	-	-	-	-	+	-	-	+	+
Geobacteraceae	+	-	-	-	-	-	-	-	+	-	-	-	-	-
Helicobacteraceae	-	-	-	-	-	-	-	-	-	+	-	-	-	-
Heliobacteriaceae	-	-	-	-	-	-	-	-	-	-	+	-	-	-
Lachnospiraceae	+	-	-	+	-	-	-	+	+	+	+	-	+	+
Lactobacillaceae	+	+	+	-	+	+	-	+	+	+	+	+	+	+
Listeriaceae	-	-	-	-	-	-	-	-	-	-	-	-	+	+
Malvaceae	-	-	-	-	-	-	-	-	-	-	-	+	+	-

Methanobacteriaceae	+	+	+	-	+	+	-	+	+	+	+	+	+	+
Methanomassiliicoccaceae	+	+	+	+	+	-	-	+	+	+	+	+	+	+
Micrococcaceae	-	-	-	-	-	-	+	-	-	-	-	-	-	-
Moraxellaceae	-	-	+	-	-	-	-	-	-	+	-	+	+	+
Neocallimastigaceae	-	-	+	-	-	-	-	-	-	-	-	-	-	-
Oscillospiraceae	-	-	+	+	-	-	-	+	+	+	+	-	+	-
Paenibacillaceae	-	-	-	-	-	-	-	+	-	+	+	-	-	-
Peptococcaceae	-	-	-	-	-	-	-	+	-	+	+	-	-	-
Peptoniphilaceae	-	-	-	-	-	-	-	+	-	+	+	-	-	-
Peptostreptococcaceae	+	-	+	-	+	+	-	+	+	+	+	+	-	-
Picobirnaviridae	-	-	-	-	-	-	-	-	-	+	+	-	-	-
Picornaviridae	-	-	-	+	-	-	+	-	+	+	+	-	-	-
Planococcaceae	-	-	-	-	-	+	-	-	-	-	+	+	-	-
Polystomatidae	-	-	-	-	-	-	+	-	-	-	-	-	-	-
Porphyromonadaceae	+	+	+	+	-	+	+	+	+	+	+	-	+	+
Prevotellaceae	+	+	+	+	+	+	-	+	+	+	+	+	+	+
Propionibacteriaceae	-	-	-	-	-	-	+	-	-	-	-	-	+	-
Pseudomonadaceae	-	+	+	-	-	+	+	-	-	-	-	+	+	+
Rhizobiaceae	-	-	-	-	-	-	+	-	-	-	-	-	-	-
Rhodobacteraceae	-	-	-	-	-	-	+	-	-	-	-	-	-	-
Rikenellaceae	-	-	-	-	-	-	+	+	-	-	-	-	-	-
Ruminococcaceae	+	+	-	+	+	+	-	+	+	+	+	-	+	+
Saccharomycetaceae	-	-	-	-	-	-	-	+	-	-	-	-	-	-
Siphoviridae	-	-	-	-	-	-	+	-	-	-	-	-	-	-
Spathidiidae	+	-	-	+	-	-	-	-	-	-	-	-	+	-
Sphingobacteriaceae	+	-	-	-	-	-	-	-	-	-	-	-	-	-
Spirochaetaceae	+	+	-	+	-	+	-	+	+	+	+	+	+	+
Spiroplasmataceae	-	-	-	-	-	-	-	-	-	-	-	-	+	-
Staphylococcaceae	-	-	-	-	-	-	+	-	-	-	-	-	-	-
Streptococcaceae	-	-	-	+	+	-	+	+	-	+	+	-	+	+
Succinivibrionaceae	+	+	-	-	-	-	-	-	-	-	-	-	-	-
Suidae	-	-	+	+	-	-	-	+	+	+	+	-	-	-
Thermoanaerobacteraceae	-	-	-	-	-	-	-	-	-	+	-	-	-	-
Thermoanaerobacterales Family III. Incertae Sedis	-	-	+	-	+	-	-	-	-	-	-	-	-	-
Veillonellaceae	+	+	-	-	+	+	-	-	+	-	+	-	+	+
Vibrionaceae	+	-	-	-	-	-	-	-	-	-	-	-	-	-
Xanthomonadaceae	-	-	+	-	-	-	-	-	-	-	-	-	-	-
